# *In Vitro* antibacterial and *in Vivo* cytotoxic activities of *Grewia paniculata*

**Published:** 2015

**Authors:** Mahmuda Nasrin, Pritesh Ranjan Dash, Mohammad Shawkat Ali

**Affiliations:** 1 *Department of Pharmacy Jahangirnagar University, Bangladesh*; 2 *Department of Pharmacy, BRAC University, Bangladesh*

**Keywords:** *Grewia paniculata*, *Malvaceae*, *Artemia salina*, *Disc diffusion method*

## Abstract

**Objectives:**

* Grewia paniculata* (Family: Malvaceae) has been used to treat inflammation, respiratory disorders and fever. It is additionally employed for other health conditions including colds, diarrhea and as an insecticide in Bangladesh. The aim of the present study was to investigate the antibacterial and cytotoxic activities of different extracts of *Grewia paniculata*.

**Materials and Methods:**

The antibacterial activity was evaluated against both gram negative and gram positive bacteria using disc diffusion method by determination of the diameter of zone of inhibition. Cytotoxic activity was performed by brine shrimp (*Artemia salina*) lethality bioassay.

**Results:**

In disc diffusion method, all the natural products (400 μg/disc) showed moderate to potent activity against all the tested bacteria. The ethanol extract of bark (EEB) and ethanol fraction of bark (EFB) (400 μg/disc) exhibited highest activity against *Shigella dysenteriae* with a zone of inhibition of 23±1.63 mm and 23±1.77 mm respectively. In the brine shrimp lethality bioassay all the extracts showed moderate cytotoxic activity when compared with the standard drug vincristin sulphate. For example, LC50 value of the ethanol fraction of bark (EFB) was 3.01 μg/ml while the LC50 of vincristine sulphate was 0.52 μg/ml.

**Conclusions:**

The results suggest that all the natural products possess potent antibacterial and moderate cytotoxic.

## Introduction

*Grewia paniculata* (Family: Malvaceae, Bengali name: Fattashi), which is a herbaceous plant is widely distributed and naturally grows throughout Bangladesh. The plant is occurring in India, Andaman and Nicobar (Andaman Islands), Sri Lanka, China, Thailand, Vietnam, Malaysia etc. Traditional beliefs claim it services the digestive system to work better and it is additionally employed for other health conditions including colds, diarrhea, hepatitis, heat stroke and dyspepsia. This plant is traditionally used in wound healing, fever and as an insecticide in Bangladesh (Ghani, 1998 ▶; Nadkarni and Basu, 1996 ▶). Pharmacological properties such as analgesic and cytotoxic activities (Rahman et al., 2011 ▶), antidiarrhoeal activities (Rahman et al., 2012 ▶), insecticidal activity against *Aedes aegypti* second instar larvae (Bandara et al., 2000 ▶) has been reported. Two new piperidine alkaloids, microcosamines A and B were isolated from leaves and showed significant larvicidal activity against *Culex quinquefasciatus *(Feng et al., 2008 ▶). As a part of our continuing studies (Nasrin et al., 2013 ▶; Dash et al., 2014b ▶) on natural products for their pharmacological properties, we investigated different extracts of *Grewia paniculata* for their antibacterial and cytotoxic activities. 

## Materials and Methods

**Collection of the plant**

The plant of *Grewia paniculata *was collected from the local area of Gazipur, Dhaka, Bangladesh in September, 2011. The collected plant was then identified by Bushra Khan, Principal Scientific Officer, Bangladesh National Herbarium, Mirpur, Dhaka and a voucher specimen has been deposited (DACB: 35,942) for further reference.

**Extraction and fractionation of the plant material**

The plant parts were extracted by a cold extraction method. The bark (1000 g) and leaf (400 g) powder were taken and soaked with 3000 ml and 900 ml of ethanol for 3 consecutive days at 25 °C. The extracts were filtered and evaporated on rotary evaporator under reduced pressure. Recovered solvent was again used for percolation for another 3 days. The process was repeated three times to obtain 132 g bark (yield 13.2%) and 16.94 g leaf (yield 4.24%) extract of *Grewia paniculata*. The bark extract was further partitioned using petroether, chloroform and ethanol. The ethanol extract of the bark (EEB), as well as petroether fraction (PEF), chloroform fraction (CHF), ethanol fraction (EFB), and ethanol extract of leaf (EEL) were subjected to antibacterial and cytotoxic activity test.

**Drugs and chemicals**

Ethanol and DMSO (dimethyl sulfooxide) were purchased from Merck, Germany. Ciprofloxacin was obtained from Oxoid Ltd., Basingstoke, England. Vinchristin sulphate was purchased from Cipla Ltd., Goa, India.

**Phytochemical screening**

All the extracts of *Grewia paniculata* were qualitatively tested for detection of carbohydrates, tannins, flavonoids, saponins, proteins, steroids, alkaloids, glycosides, glucosides and resins following standard phytochemical procedures (Ghani, 2003 ▶).

**Test organisms**

The bacterial species of gram positive and gram negative used in the present study were *Staphylococcus aureus *(*S. aureus*), *Bacillus cereus (B. cereus) *and *Escherichia coli *(*E. coli*), *Pseudomonas aureus* (*P. aureus*), *Shigella dysenteriae* (*S. dysenteriae*), *Klebsiella pneumoniae* (*K. pneumonia*). These were collected as pure culture from the Institute of Nutrition and Food Sciences (INFS), University of Dhaka, Dhaka. Simple zoological organism *Artemia salina* was used for cytotoxicity study.

**Antibacterial screening**

*Disc diffusion method*

*In vitro *antibacterial screening of all the extracts was carried out by the disc diffusion method (Bauer et al., 1966 ▶). All the bacterial strains were grown and maintained on Muller Hinton agar (Hi media, India) media at 37 ^○^C and pH (7.3±0.2). The bacteria were sub-cultured overnight in Muller Hinton broth, which was further adjusted to obtain turbidity comparable to McFarland (0.5) standard when required (Sein et al., 2008 ▶). The test microbes were taken from the broth culture with inoculating loop and transferred to test tubes containing 10.0 mL sterile distilled water. The inoculums were added until the turbidity was equal to 0.5 McFarland standards. Cotton swab was then used to inoculate the test tube suspension onto the surface of Muller Hinton agar and the plate was allowed to dry. Sterilized Whatman paper discs (6mm in diameter) were treated with 10 μl of a solution of 40 mg/ml of the extracts using a micropipette and dried in air under aseptic condition and placed at equidistance in a circle on the seeded plate. The extracts were dissolved separately in ethanol and applied to sterile discs at a concentration of 400 μg/disc (Dash et al., 2014a ▶; Abdul et al., 2010 ▶; Yasmin et al., 2009 ▶) and carefully dried to evaporate the residual solvent. Here, ciprofloxacin 5 μg/disc (Dash et al., 2014a ▶; Parvez et al., 2005 ▶) was used as the standard. These plates were then kept at low temperature (4 ºC) for 4-6 hours to allow maximum diffusion of the test materials and standard drugs. The plates were then incubated at 37 ºC for 24 hours to allow maximum growth of the organisms. The test material having antibacterial activity inhibited the growth of the microorganisms and a clear, distinct zone of inhibition was visualized surrounding the discs. The antibacterial activity of the test agents was determined by measuring the diameter of zone of inhibition expressed in mm. The antibacterial activity was classified as potent active (>18 mm), moderate active (14–17 mm), and mild active (11-13 mm) and less than 8 mm was considered as inactive. 

**Cytotoxicity studies**

*Brine shrimp lethality bioassay*

Brine shrimp lethality bioassay (Meyer et al., 1982 ▶) was carried out to investigate the cytotoxicity of the extracts of medicinal plants of Bangladesh. Brine shrimps (*Artemia salina*) were hatched using brine shrimp eggs in a conical shaped vessel (1L), filled with sterile artificial seawater (prepared using sea salt 38 g and adjusted to pH 8.5 using 1N NaOH) under constant aeration for 48 hr. After hatching, active nauplii free from egg shells were collected from brighter portion of the hatching chamber and used for the assay. Ten nauplii were drawn through a glass capillary and placed in each vial containing 4.5 ml of brine solution. In each experiment, 0.5 ml of the plant extract was added to 4.5 ml of brine solution and maintained at room temperature for 24 hr under the light and surviving larvae were counted. Experiments were conducted along with control (vehicle treated), different concentrations (1.25 to 320 μg/ ml) of the test substances in a set of two tubes per dose. Vincristine sulfate (0.156 to 40 μg/ ml) was used as a positive control in the bioassay (Parvin et al., 2010 ▶; Abdul et al., 2010 ▶). The mean percentage mortality was plotted against the logarithm of concentrations and the concentration killing fifty percent of the larvae (LC_50_) was determined from probit analysis described by Finney (1971) as well as linear regression equation using the software “Microsoft Excel – 2003”**. **

**Statistical analysis**

All assays were performed in triplicate under strict aseptic conditions to ensure consistency of all findings. Data of all experiments were statistically analyzed and expressed as the mean ± SEM of three replicate experiments.

## Results

**Phytochemical Screening**

Preliminary phytochemical group tests revealed that the extracts of *Grewia paniculata* contain carbohydrates, tannins, flavonoids, saponins, proteins, steroids and alkaloids (Table 1). 

**Antibacterial activity**

Results of antibacterial activity are presented in Table 2. EEB, CHF and EEL (400 μg/disc) showed moderate to potent activity against *Staphylococcus aureus, Escherichia coli, Pseudomonas aureus* and *Shigella dysenteriae* respectively. On the other hand, PEF and EEL exhibited potent activity against *Escherichia coli *and *Shigella dysenteriae* respectively. CHF was inactive against* Bacillus cereus* and EEB was inactive against *Klebsiella pneumoniae.*Both *Bacillus cereus* and *Klebsiella pneumoniae* were not inhibited by PEF and EEL. The EEB and EFB (400 μg/disc) exhibited highest activity against *Shigella dysenteriae* with a zone of inhibition of 23±1.63 mm and 23±1.77 mm respectively. 

**Table 1:** Results of phytochemical screening

^Test^	^EEB^	^PEF^	^CHF^	^EFB^	^EEL^
^Carbohydrates^	^+^	^-^	^+^	^+^	^-^
^Tannins^	^+^	^+^	^+^	^+^	^+^
^Flavonoids^	^+^	^+^	^-^	^+^	^-^
^Saponins^	^+^	^-^	^-^	^-^	^-^
^Proteins^	^+^	^-^	^-^	^-^	^+^
^Steroids^	^+^	^-^	^-^	^+^	^+^
^Alkaloids^	^+^	^+^	^+^	^+^	^+^
^Glycosides^	^-^	^-^	^-^	^-^	^-^
^Glucosides^	^-^	^-^	^-^	^-^	^-^
^Resins^	^-^	^-^	^-^	^-^	^-^

(+) =Presence, () =Absence, EEB= Ethanol extract of bark, PEF= Petroether fraction of bark, CHF= Chloroform fraction of bark, EFB= Ethanol fraction of bark, EEL= Ethanol extract of leaf

**Table 2:** Antibacterial activity of different extracts of *Grewia paniculata*

^Microorganism^	^Determination of zone of inhibition in mm^					
	^Cipro 5 μg/disc^	^EEB 400 μg/disc^	^PEF 400 μg/disc^	^CHF 400 μg/disc^	^EFB 400 μg/disc^	^EEL 400 μg/disc^
^Gram positive^						
^Staphylococcus aureus^	^24±1.22^	^19±0.81^	^10±0.81^	^18±0.41^	^8±0.41^	^18±0.81^
^Bacillius cereus^	^14±0.41^	^9±0.41^	^0^	^0^	^8±0.71^	^0^
^Gram negative^						
^Escherichia coli^	^24±0.81^	^16±1.10^	^20±1.22^	^17±1.10^	^23±1.22^	^14±0.41^
^Pseudomonas aureus^	^24±0.71^	^18±1.87^	^9±0.41^	^18±0.81^	^10±0.41^	^15±1.22^
^Shigelladysenteriae^	^23±1.63^	^23±1.63^	^18±1.22^	^15±1.63^	^23±1.77^	^14±1.41^
^Klebsiella pneumonia^	^14±1.47^	^0^	^0^	^10±1.22^	^9±0.71^	^0^

Values of the observed diameter zone of inhibition (mm). Incubation conditions for bacteria- 24 hours at 37 ^o^C. The assay was performed in triplicate and the results are the mean of three values ± SEM. EEB =Ethanol extract of bark, PEF= Petroether fraction of bark, CHF=Chloroform fraction of bark, EFB=Ethanol fraction of bark and EEL=Ethanol extract of leaf, Cipro=Ciprofloxacin, 0=No zone of inhibition, a diameter less than 8 mm was considered inactive.

**Cytotoxic activity**

*Brine Shrimp Lethality Bioassay*

The degree of lethality shown by the extracts was found to be directly proportional to the concentration of the extract ranging from the lowest concentration (1.25 μg/ml) to the highest concentration (320 μg/ml) (Table 3). The EFB was found to be maximally toxic to brine shrimp nauplii, having LC_50 _values of 3.01 μg/ml while the LC_50 _of the reference anticancer drug vincristine sulphate was 0.52 μg/ml (Table 4). The rate of mortality of the nauplii found to be increased with increasing concentration of the sample (Table 3).

**Table 3:** Effects of different extracts of *Grewia paniculata *on brine shrimp lethality test in *Artemia salina*

S**ample conc.** **(μg/ml)**	**Log conc.**	**No.ofnauplii taken**	**Average no. of nauplii dead**					**Percent of mortality**					**Vincristine Sulfate**				
			**EEB**	**PEF**	**CHF**	**EFB**	**EEL**	**EEB**	**PEF**	**CHF**	**EFB**	**EEL**	**Std.Conc.** **(μg/ml)**	**Log conc.**	** No .of ** **nauplii taken**	**No.of** **nauplii dead**	** % of** ** mortality**
**1.25**	0.090	10	3	3	3	4	3	30	30	30	40	30	0.156	-0.806	10	3	30
**2.5**	0.39	10	4	4.5	3.5	5	4	40	45	35	50	40	0.312	-0.505	10	4	40
**5**	0.69	10	4	5	5.5	6	4	40	50	55	60	40	0.625	-0.204	10	5	50
**10**	1	10	5	5.5	5.5	6.5	5	50	55	55	65	50	1.25	0.096	10	6	60
**20**	1.30	10	6.5	6	5.5	6.5	6	65	60	55	65	60	2.5	0.397	10	8	80
**40**	1.60	10	6.5	6.5	6	6.5	6.5	65	65	60	65	65	5	0.698	10	9	90
**80**	1.90	10	7.5	6.5	7	8	6.5	75	65	70	80	65	10	1	10	10	100
**160**	2.20	10	8.5	8	7.5	10	8	85	80	75	100	80	20	1.310	10	10	100
**320**	2.50	10	9	8.5	8	10	8	90	85	80	100	80	40	1.602	10	10	100

EEB=Ethanol extract of bark, PEF= Petroether fraction of bark, CHF=Chloroform fraction of bark, EFB=Ethanol fraction of bark and EEL=Ethanol extract of leaf

**Table 4:** Results of different extracts of *Grewia paniculata *on *Artemia salina*

Sample	LC_50 _(g/ml)	Regression equation	R^2^
**EEB**	**7.94**	**y = 25.46x+26.86**	**0.979**
**PEF**	**6.76**	**y = 20.20x+33.15**	**0.956**
**CHF**	**8.5**	**y = 19.65+31.65**	**0.931**
**EFB**	**3.01**	**y = 23.80x+38.47**	**0.913**
**EEL**	**9.5**	**y = 21.31x+28.93**	**0.968**
**Vincristine sulphate**	**0.524**	**y = 32.61x+59.22**	**0.942**

EEB=Ethanol extract of bark, PEF= Petroether fraction of bark, CHF=Chloroform fraction of bark, EFB=Ethanol fraction of bark and EEL=Ethanol extract of leaf.

## Discussion

Antibacterial activity of ethanolic and partially extracted products of *Grewia paniculata *was examined and found to exhibit moderate to potent antibacterial activity at 400 μg/disc dose level against gram positive and gram negative organisms which has been depicted in the (Table 2).

Preliminary phytochemical group tests revealed that the extracts of *Grewia paniculata* contain carbohydrates, tannins, flavonoids, saponins, proteins, steroids and alkaloids (Table 1). Some of the phytochemical compounds e.g. glycoside, saponin, tannin, flavonoids, terpenoid, alkaloids, have been variously reported to have antimicrobial activity (Okekeet al., 2001 ▶; Ebi and Ofoefule, 1997 ▶).

The extract of this plant also possesses alkaloidal substances which have biological activities. The presence of flavonoids, tannins and alkaloids in the extract may be responsible for activity against these microorganisms. In our study, some of the bacterial strains did not respond to crude extracts, whereas the fractions showed broad-spectrum activity against multiple strains. This might be due to masking of antibacterial activity by the presence of some inhibitory compounds or factors in the extract or synergism by the presence of some compounds or factors in the extract (Choudhury et al., 2005 ▶). The variation of antibacterial activity of our extracts might be due to distribution of antimicrobial substances, which varied from fraction to fraction of the crude extract. Similar observations were made by Vlachos et al.(1997) ▶ who found that fractionation of crude extracts tested enhanced their activity against both gram negative as well as the resistant gram positive pathogens. Brine shrimp lethality bioassay is a rapid and comprehensive bioassay for the bioactive compound of the natural and synthetic origin (McLaughlin et al., 1998 ▶). By this method, natural product extracts, fractions as well as the pure compounds can be tested for their bioactivity. In this method, *in vivo *lethality in a simple zoological organism (Brine shrimp nauplii) is used as a favorable monitor for screening and fractionation in the discovery of new bioactive natural products. This bioassay indicates cytotoxicity as well as a wide range of pharmacological activities such as antimicrobial, antiviral, pesticidal and anti-tumor etc. of the compounds (Meyer et al., 1982 ▶). The degree of lethality was directly proportional to the concentration of the extract ranging from significantly with the lowest concentration (1.25μg/ml) to highly significant with the highest concentration (320µg/ml). Maximum mortalities took place from the concentration 320µg/ml, whereas least mortalities were at 1.25 µg/ml concentration. In other words, mortality increased gradually with the increase in concentration of the test samples (Table 3). In brine shrimp lethality bioassay, the EEB, PEF, CHF, EFB and EEL exhibited moderate cytotoxic activity whose LC_50 _values were 7.94, 6.76, 8.5, 3.01 and 9.5 μg/ml respectively whereas the LC_50_ value of vincristine sulfate was 0.52 μg/ml (Table 4). So, it can be well predicted that the crude ethanolic extract and its different partitioning fractions possess cytotoxic principles and have considerable cytotoxic potency. 

Overall, the plant extract possesses moderate to potent antibacterial and moderate cytotoxic effects and may have potential bioactive principles. While the brine shrimp lethality assay may be inadequate for elucidation of mechanism of action, yet is a convenient way of monitoring biological activities of plant used in traditional medicine. The result of this study justifies the use of the *Grewia paniculata *by traditional healers in Bangladesh. Our results indicate the plant extract to be moderately toxic. There is need for further studies on this plant to ascertain the active compound(s) and its true toxicity so as to maximize the widely used medicinal plant in development of antibacterial drugs. 
